# Adiponectin Increase in Patients Affected by Chronic Obstructive Pulmonary Disease with Overlap of Bronchiectasis

**DOI:** 10.3390/life13020444

**Published:** 2023-02-04

**Authors:** Ersilia Nigro, Marco Mosella, Aurora Daniele, Marta Mallardo, Mariasofia Accardo, Andrea Bianco, Fabio Perrotta, Filippo Scialò

**Affiliations:** 1 CEINGE, Biotecnologie Avanzate Scarl, 80145 Napoli, Italy; 2 Dipartimento di Scienze e Tecnologie Ambientali, Biologiche, Farmaceutiche, Università della Campania “Luigi Vanvitelli”, 81100 Caserta, Italy; 3 Istituti Clinici Scientifici Maugeri IRCCS, Pulmonary Rehabilitation Unit of Telese Terme Institute, 82037 Telese Terme, Italy; 4 Dipartimento di Medicina Molecolare e Biotecnologie Mediche, “Federico II” Università degli Studi di Napoli, 80131 Napoli, Italy; 5 Department of Translational Medical Sciences, University of Campania L. Vanvitelli, 80138 Naples, Italy

**Keywords:** adiponectin, biomarker, chronic obstructive pulmonary disease, bronchiectasis, pro-inflammatory cytokines

## Abstract

Chronic obstructive pulmonary disease (COPD) is characterized by respiratory symptoms and non-reversible airflow limitation with recurrent episodes of acute exacerbations. The concurrent presence of bronchiectasis in patients with COPD is associated with reduced respiratory function as well as increased exacerbation risk. Adiponectin is a promising biomarker in COPD, as greater high molecular weight (HMW) oligomer levels have been observed among COPD patients. Here, we investigate adiponectin levels in two groups of COPD patients characterized by the presence or absence of bronchiectasis (BCO), comparing both groups to healthy controls. We evaluated serum adiponectin levels in COPD patients, those with BCO, and healthy subjects and characterized the pattern of circulating adiponectin oligomers. We found that forced volume capacity % (FVC%) and forced expiratory volume % (FEV1%) were lower for BCO patients than for COPD patients. COPD patients had higher levels of adiponectin and its HMW oligomers than healthy controls. Interestingly, BCO patients had higher levels of adiponectin than COPD patients. We showed that expression levels of IL-2, -4, and -8, IFN-γ, and GM-CSF were significantly higher in BCO patients than in healthy controls. Conversely, IL-10 expression levels were lower in BCO patients. Our data suggest that the increased levels of adiponectin detected in the cohort of BCO patients compared to those in COPD patients without bronchiectasis might be determined by their worse airway inflammatory state. This hypothesis suggests that adiponectin could be considered as a biomarker to recognize advanced COPD patients with bronchiectasis.

## 1. Introduction

Chronic obstructive pulmonary disease (COPD) is a chronic inflammatory condition characterized by persistent respiratory symptoms and non-reversible flow limitation caused by chronic exposure to smoking or other noxious gases [1]. COPD is characterized by episodes of exacerbations that are mainly induced by viruses, bacterial infection, pollution, or hemodynamic causes and defined by an acute worsening in respiratory symptoms requiring changes in treatment or hospitalization [2]. Exacerbations have been linked to disease-associated morbidity and mortality, placing significant strain on medical facilities, increasing resource burden, and driving up ongoing healthcare costs. Adult bronchiectasis is characterized by irreversible dilation of the bronchial tree with persistent cough and sputum production, leading to inflammation and progressive lung damage that results ultimately in respiratory failure, lower quality of life, and increased mortality [3,4]. Recently, literature data reported a high annual prevalence of bronchiectasis (701 per 100,000 persons) with over 20,000 patients being hospitalized for respiratory infections [5], highlighting that bronchiectasis is a growing healthcare problem [6]. The heterogeneous nature of adult bronchiectasis likely contributes to the poor results seen thus far from clinical trials [6,7]. 

Bronchiectasis frequently co-exists with COPD; however, whether the concurrent presence of these two disorders represents a definite COPD phenotype has not been fully elucidated [8]. Quite often, bronchiectasis is an incidental finding on computed tomography (CT) scans and may be subclinical and have unrecognized effects beyond respiratory failure, which clinicians and researchers need to address to improve prognosis [9]. In particular, an understanding of increased cardiovascular risk in patients with bronchiectasis is emerging [10], emphasizing the need for the identification of new biomarkers to improve early diagnosis and prognosis of COPD exacerbations. 

Adipose tissue, for a long time considered the only primary site of storage for excess energy, is a complex, essential, and highly active metabolic and endocrine organ. In recent years, adipose tissue has been reported to be involved in systemic inflammation in COPD patients through the secretin of bioactive hormones, known as adipokines, mediators involved in the regulation of metabolic and inflammatory processes. Adiponectin is one of these adipokines that has important anti-inflammatory, anti-atherosclerotic, and anti-obesity effects [11]. Adiponectin normally circulates at quite high levels, such as 5–30 μg/mL. There is great interest in the structure of adiponectin since its oligomeric state may specify its biological activities; indeed, it circulates in serum as trimers (LMW), hexamers (MMW), and high molecular weight (HMW) complexes. Epidemiological data indicate that the HMW oligomers are the most biologically relevant form [12]. In metabolic disorders, adiponectin expression is strongly downregulated and inversely associated with body mass index (BMI) and glucose and cholesterol levels. We and other researchers previously reported that adiponectin is an important serum biomarker in COPD [13,14,15] since its serum levels are higher in COPD patients than in healthy controls. In addition, adiponectin increase is strictly and specifically associated with a significant increase in levels of HMW oligomers, suggesting that the control of adiponectin concentrations reflects a functional role of this adipokine in COPD [13]. Notably, adiponectin has been associated with increased risk of respiratory mortality in COPD but is not significantly influenced by smoking status; thus, it is a very promising biomarker of cardiovascular outcomes in COPD [16]. 

To our knowledge, there are no studies investigating adiponectin expression in patients with bronchiectasis-COPD overlap (BCO). Therefore, the aim of this study was to analyze adiponectin involvement in the worsening of COPD toward BCO. To this aim, we analyzed adiponectin levels in two recruited groups of COPD patients characterized by the presence or absence of bronchiectasis, comparing both groups with healthy controls. Furthermore, we measured and compared different key cytokines for pulmonary health, such as interleukin (IL)-2, IL-6, IL-8, IL-10, granulocyte-macrophage colony-stimulating factor (GM-CSF), and interferon-γ (IFN-γ) in BCO patients, COPD patients, and healthy controls.

## 2. Materials and Methods

### 2.1. Recruitment of Patients

A group of 30 BCO patients (15 males, 15 females; age 60 ± 15.9 years) and 29 COPD patients (16 males, 13 females; age 61 ± 8.4 years) were recruited from the Department of Translational Medical Sciences, University of Campania “Vanvitelli”, Italy. 

As controls, 29 age- and sex-matched healthy volunteers (14 males, 15 females; age 57 ± 10 years) were recruited from the staff of CEINGE—Biotecnologie Avanzate (Naples, Italy). Serum samples were collected at hospital admission and stored at −80 °C for subsequent analysis. The research was performed in accordance with the Declaration of Helsinki and approved by the local Ethical Committee A.O. dei Colli—Università della Campania “L. Vanvitelli”. Written informed consent was obtained from all participants before starting the study. 

COPD patients were required to fulfill the following eligibility criteria: (1) age ≥ 40 years; (2) smokers or former smokers with pack years ≥ 10; (3) post-bronchodilator forced expiratory volume in 1s (FEV1) divided by the forced volume capacity (FVC) with a value of less than 0.70 indicating a persistent expiratory airflow limitation according to the Global Initiative for Chronic Obstructive Lung Disease (GOLD) 2021 report [17]; (4) BMI > 20 to exclude the main source of bias and overfitting of statistical models. 

Bronchiectasis in COPD patients was diagnosed in the presence of both bronchial dilations on CT and clinical symptoms, such as cough, sputum production, and/or recurrent respiratory infection in adults according to the Europe Respiratory Society and British Thoracic Society guidelines [18,19]. The clinical phenotype of bronchiectasis with COPD overlap was confirmed by spirometry and CT scan. Exclusion criteria for both COPD and BCO included: previous diagnosis of metabolic syndrome; history of any type of cancer other than non-melanoma skin cancer; and use of antibiotics or systemic glucocorticosteroids during the previous 12 weeks. The degree of severity of COPD was established using the values of the FEV1% predicted, history of exacerbations, and severity of symptoms by the COPD Assessment Test (CAT) and modified Medical Research Council (mMRC) questionnaire according to the GOLD recommendations. A stable state was defined as the absence of significant changes in symptoms beyond the expected daily variation, requiring treatment changes during the previous 12 weeks. Airflow limitation severity was stratified as follows: GOLD 1 (mild, FEV1 ≥ 80% predicted), GOLD 2 (moderate, 50% ≤ FEV1 < 80% predicted), GOLD 3 (severe, 30% ≤ FEV1 < 50% predicted), and GOLD 4 (very severe, FEV1 < 30% predicted) [17]. 

### 2.2. Anthropometric and Biochemical Measurements

We recorded height, weight, and BMI. Anthropometric measurements were performed in triplicate according to Cameron [20]. Body weight, expressed in kilograms, was measured at fasting state in the morning with a mechanical balance (± 0.1 kg, SECA 700, Hamburg DE). BMI was calculated as body weight divided by height squared (kg/m^2^) with categories in accordance with the WHO guidelines. Blood samples from all participants were collected in the morning after an overnight fast at the time of sampling for routine purposes. Serum samples were separated and analyzed as follows: serum albumin was measured by colorimetric method (Albumin BCG assay; Abbott Diagnostics, Rome, Italy); glucose, total cholesterol, and triglyceride levels were analyzed using specific enzymatic assays; and HDL and LDL cholesterol levels were measured using Ultra HDL methods and Multigent Direct LDL, respectively (Abbott Diagnostics, Rome, Italy). All assays were performed using an automated biochemistry analyzer (Architect ci16200 Integrated System; Abbott Diagnostics, Rome, Italy). 

### 2.3. Measurement of Total and HMW Adiponectin by ELISA Assay

Total serum adiponectin levels were measured by enzyme-linked immunosorbent assay (ELISA), as previously reported [13]. A calibration curve was constructed using human recombinant adiponectin (Biovendor R&D, Brno, Czech Republic) as the standard. The amount of HMW adiponectin was evaluated using a commercially available ELISA kit according to the manufacturer’s instructions (Biovendor R&D, Brno, Czech Republic). Each serum sample was tested three times in duplicate.

### 2.4. Measurement of Cytokine Levels by ELISA Assay

Levels of IL-2, IL-4, IL-6, IL-8, IL-10, IFN-γ, and GM-CSF were measured in BCO patients vs. healthy controls using a commercially available kit (Bio-Plex Pro™ Human Cytokine 8-plex Assay; Bio-Rad, Hercules, CA, USA). The assay was performed according to the manufacturer’s instructions and the concentrations of cytokines were calculated by comparing reads with a 5-parameter logistic standard curve using a Bioplex-200 instrument (Bio-Rad, Hercules, CA, USA). The sensitivity of each dosable cytokine is reported in Supplementary Table S1. Each serum sample was assayed three times in duplicate.

### 2.5. Western Blotting Analysis

Sera were quantified for total proteins using the Bradford method (Bio-Rad, Hercules, CA, USA) and 10 µg of total proteins were treated with 1× Laemmli buffer, heated at 95 °C for 2 min, separated on 10% SDS-PAGE gel, and transferred, as previously described [21]. The blots were scanned using the ChemiDoc MP imaging system (Bio-Rad, Hercules, CA, USA) and analyzed by densitometry using ImageJ software (http://rsbweb.nih.gov.ij/, accessed on 1 September 2022). Each sample was tested three times in duplicate.

### 2.6. Statistical Analysis

Continuous variables are given as the mean and standard deviation and categorical variables are given as absolute and relative frequencies. The univariate analysis was performed using parametric (Student’s *t*-test for independent samples) and non-parametric statistics (U-Mann Whitney test) for continuous variables. Fisher’s exact test was performed for categorical variables. The potential association between higher and low levels of adiponectin in determining clinical outcomes in patients was assessed using the univariate model. Odds ratios (ORs) with 95% confidence intervals (CIs) were estimated using a logistic regression model with the significant variables of the univariate model and disease duration as covariates. Sample size calculation was based on the dependent continuous variable (adiponectin) in three different groups. A total of 88 patients was included, based on the assumption of the standard deviation of the dependent variable (power 0.8 alfa 0.05). Multiple non-parametric comparisons were made using ANOVA. Analysis was performed using IBM SPSS Statistical software version 21.0. (IBM Corp., Armonk, NY, USA). A *p*-value of <0.05 was considered statistically significant.

## 3. Results

### 3.1. Anthropometric and Biochemical Characteristics of Patients and Controls

The anthropometric and biochemical characteristics of the patients are reported in Table 1. A total of 29 COPD patients without bronchiectasis, 30 BCO patients, and 29 healthy controls were recruited for the study. The three studied populations were similar in age, BMI, and sex distribution. Smoking status was different between groups, as COPD and BCO patients were current or former smokers while the control group included only non-smokers. No significant difference was observed in the smoking history between COPD and BCO patients (*p* = 0.08). Pack years were not different between COPD and BCO patients (*p* = 0.82). Biochemical and anthropometrical parameters were considered; FVC% and FEV1% were lower in BCO patients than in COPD patients without bronchiectasis.

### 3.2. Adiponectin Is Differently Expressed in COPD Patients with and without Bronchiectasis and in Comparison, to Healthy Controls

As shown in Table 2, adiponectin levels among the 3 studied groups (COPD, BCO, and controls) were statistically different. Our data showed that COPD patients had statistically higher levels of adiponectin than healthy controls (*p* < 0.001). Interestingly, we found that BCO patients had higher levels of serum adiponectin than COPD patients without bronchiectasis (*p* < 0.001) (Table 2), suggesting a different regulation of adiponectin expression in exacerbated patients.

Next, we verified whether the differences in adiponectin values were influenced by confounding factors. As shown in Table 3, the statistical analysis indicated that adiponectin level modulation was not influenced by BMI (*p* = 0.80), glycemia (*p* = 0.23), total cholesterol (*p* = 0.25), and triglycerides (*p* = 0.17), suggesting that adiponectin modulation was related to the pulmonary alteration rather than to the metabolic confounding parameters of the patients.

Furthermore, we explored the possible correlation between adiponectin levels and airflow limitation severity in BCO patients stratified according to FEV1. As shown in Supplementary Figure S1, airflow limitation severity (mild, moderate, severe) was not significantly correlated with adiponectin serum level (*p* = 0.728).

### 3.3. Adiponectin Oligomeric State 

**Figure 1:**
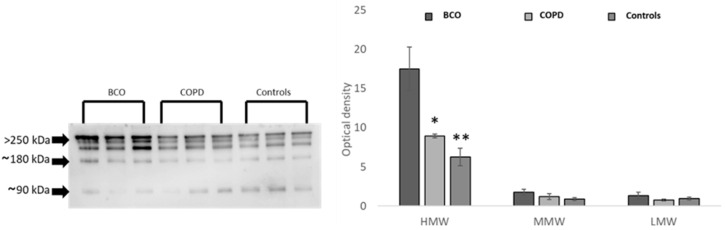
Oligomeric distribution of adiponectin in the 3 studied populations: COPD, BCO, and controls. * *p* < 0.05, ** *p* < 0.01. The uncropped blots are shown in Figure S2.

Finally, we explored whether specific adiponectin oligomeric forms (HMW, MMW, LMW) were responsible for adiponectin upregulation in BCO patients compared to COPD patients and healthy controls (Figure 1). Levels of HMW, the most biologically active oligomers, were higher in BCO patients than in COPD patients and in controls, suggesting that the adiponectin regulation represents a functional response of the adipose tissue to the pulmonary injuries established in COPD and BCO diseases. MMW and LMW oligomers were not differently modulated in the three groups of subjects (Figure 1).

### 3.4. Cytokines Concentration in BCO Patients and Healthy Controls

To better understand the inflammatory milieu, we measured and compared the expression of different cytokines (IL-2, IL-6, IL-8, IL-10, GM-CSF, IFN-γ) in 30 BCO patients, 29 COPD patients, and 29 healthy controls. The results are shown in Table 4. IL-6, IL-4, IL-8, IFN-γ, and GM-CSF were expressed at significantly higher levels in both COPD and BCO patients than in healthy controls. Interestingly, levels of the same inflammatory markers were further increased in BCO patients compared to those in COPD patients. On the contrary, IL-10 levels were significantly lower in both groups of patients with respect to those in the healthy controls.

## 4. Discussion

COPD is an increasingly important cause of morbidity, disability, and mortality worldwide. Disease progression of COPD is variable, with some patients having a stable course while others suffering progression to severe breathlessness, respiratory failure, and death. Bronchiectasis in COPD is a challenging disease that carries a heavy healthcare burden and significant mortality and morbidity. Bronchiectasis and COPD overlap is associated with poorer outcomes and increased mortality than either disease alone [22]. Bronchiectasis, which is characterized by abnormal and irreversible distortion of the bronchi, increases the rate of infections and hospital admissions for COPD patients [6,7]. The challenge in the management of bronchiectasis is to prioritize the identification of biomarkers able to discern progression and severity in COPD patients. In this scenario, the etiology of bronchiectasis is considered to greatly influence its pathophysiology and the involvement of inflammatory and immune mediators can be highly variable. Therefore, although there have been some advances in understanding the molecular mechanisms underlying disease progression in the last ten years, there is still an unmet need to identify biomarkers able to determine the progression and presence of overlapping pathologies. 

A crosstalk between adipose tissue and the lung has been suggested, with a possible contribution of adipose tissue in the control of the lung inflammatory state typically established in COPD. Potential links between adipose tissue and COPD inflammation/metabolic derangements might be related to the endocrine functions of adipose tissue and therefore, the secretion of adipokines. To our knowledge, this is the first study to analyze adiponectin levels in COPD patients with concurrent presence of bronchiectasis. We found an increase in adiponectin levels of bronchiectasis-COPD patients compared to those without bronchiectasis, regardless of airflow limitation severity. These data suggest that adiponectin might provide a serum biomarker of bronchiectasis in COPD patients, although the molecular basis for such upregulation is far from being clarified. 

In this context, we speculate that adiponectin regulation might be traced back to two possible biological events: the former could be correlated to the interaction between adiponectin and the body composition of patients, which seems to be influenced by the co-existence of bronchiectasis; the latter could be due to the inflammatory state of patients exacerbated by exalted chronic inflammation, recurrent acute respiratory infections, or bacterial colonization of the airway. 

Following the first hypothesis, adiponectin regulation might represent a metabolic response in bronchiectasis-COPD patients that very often undergo a fat-free mass depletion [22] rather than a response to pulmonary function decline. In this scenario, a limitation of our study is the lack of data regarding the body composition of patients. However, in support of our data and speculations, Oliveira et al. reported that adiponectin levels were significantly and positively correlated with fat mass and the fat mass index and negatively correlated with fat-free mass, the fat-free mass index, and hand dynamometry in patients with bronchiectasis of any etiology [22]. On the contrary, a previous study reported that adiponectin level was associated with lung function decline in a COPD cohort and the presence of emphysema was associated with higher plasma levels of adiponectin [23]. Previously, we reported higher levels of adiponectin in COPD with a specific increase in levels of HMW oligomers [13], suggesting a functional regulation of adiponectin expression in COPD. Here, we found a further increase in levels of HMW oligomers in BCO patients, sustaining the hypothesis that adiponectin acts as an inflammatory modulating molecule subjected to differential regulation of its expression according to the lung disease. To our knowledge, there are no published data about HMW oligomers in bronchiectasis, both COPD-determined and not.

Sustained inflammation appears to play a major role in both respiratory complications and bronchiectasis exacerbations in COPD patients and our data suggest adiponectin as one of the inflammatory cytokines involved. Previously, we reported that adiponectin level was inversely associated with the neutrophil-to-lymphocyte ratio in COPD, an observation indicating an anti-inflammatory action of this adipokine in COPD [24]. In addition, in support of this data, in vitro studies evidenced that adiponectin decreased pro-inflammatory cytokine production in epithelial cells and alveolar macrophage polarization [25,26]. Thus, the anti-inflammatory activity of adiponectin might contribute to its therapeutic potential in airway inflammation [27]. In light of this evidence, the increased levels of adiponectin in our bronchiectasis-COPD cohort compared to those in patients without bronchiectasis might be determined by their worse airway inflammatory state. 

Inflammation is a key component of many common respiratory disorders, including asthma, COPD, bronchiectasis, and acute respiratory distress syndrome [28,29,30]. Chronic inflammation plays a central role in bronchial injury, inducing an exacerbated immune response [31] that is characterized by increased release of pro-inflammatory cytokines such as IL-6 and IL-8 [32]. Previous studies have demonstrated that increased cytokine levels in bronchoalveolar lavage fluid are a hallmark of bronchiectasis, whereas contrasting results have been reported in plasma [33]. In accordance with these data, here we found that IL-4, IL-8, IFN-γ, and GM-CSF were expressed at significantly higher levels in the sera of BCO and COPD patients than in healthy controls. Moreover, we found a further increase in IFN-γ, GM-CSF, IL-8, and IL-6 levels in BCO patients compared those in COPD patients, suggesting that airflow obstruction could play a role in worsening the inflammatory process. In accordance with our data, Uzeloto et al. evaluated the expression of intracellular cytokines in CD4+ T lymphocytes, finding that individuals with greater bronchial obstruction presented a higher proportion of IL-8 [34]. On the contrary, IL-10 levels were lower in our cohort of patients than in healthy subjects.

IL-10 acts as an anti-inflammatory agent that inhibits the activation of T lymphocytes, thus resulting in decreased production of pro-inflammatory cytokines. In a previous study, increased plasma levels of the immunomodulatory and anti-inflammatory cytokine IL-10 were found in adults with bronchiectasis compared to those without bronchiectasis [35]. The discrepancy with our results might be linked to the different study populations: our patients had a phenotype of COPD with concurrent bronchiectasis. In COPD patients, lower levels of IL-10 have been previously reported, suggesting that bronchiectasis is not influenced by this cytokine’s dysregulation typical of COPD disease.

IL-2 is a crucial cytokine for pulmonary diseases and is associated with COPD. Recently, Zhang et al. demonstrated in a cohort of 315 patients that patients with adverse outcomes had higher concentrations of serum soluble IL-2 receptor than patients with good outcomes, which was negatively correlated with pulmonary function, suggesting that an elevated soluble IL-2 receptor level is a predictor for the risk of adverse outcomes in COPD [36]. We found that serum IL-2 levels of BCO patients were considerably lower than those in healthy controls, confirming that this cytokine was involved in this group of patients. IL-6 is a pro-inflammatory cytokine extensively studied in COPD and pulmonary diseases and associated with a worse prognosis [37]. Furthermore, besides having increased levels in stable COPD patients, IL-6 may be used as a predictor of exacerbation [38]. Finally, IL-4 has been shown to be involved in the differentiation of CD4+ T cells into TH2 cells and in the activation of myeloid cells, resulting in the stimulation of wound healing and suppression of harmful over-inflammation [39]. In accordance with our data, IL-4 has already been shown to be upregulated in COPD patients and to correlate with disease severity [40]. Interestingly, we showed here that IL-4 levels were decreased in our BCO population. A previous study already demonstrated that IL-4 expression was also downregulated in patients with asthma and COPD overlap (ACO), proposing IL-4 as a biomarker to distinguish between asthma and ACO. In the same way, our study results suggest that IL-4 could be used to discern between COPD and BCO patients. 

Increased concentrations of IFN-γ have been suggested to predict the exacerbation of COPD, and, accordingly, IFN-γ levels were considerably elevated in our cohort of BCO patients [41]. Recently, although still under investigation, IFN therapy has been proposed to prevent COPD exacerbations [42]. Therefore, the interplay between adipocytokines and pro-inflammatory mediators might play a central role both in acute exacerbation triggered by viral and bacterial pathogens [43,44]. 

GM-CSF has been implicated as an important mediator in the pathogenesis of asthma and COPD. Although the expression of GM-CSF and its receptor in airway samples from COPD patients with differing disease severity needs to be further explored, its overexpression in the sputum of COPD patients showed a clear association with disease severity [45]. Accordingly, here we found a considerable increase in serum GM-CSF levels of BCO patients.

However, it is worth mentioning that contrasting results have been found regarding inflammatory cytokines, with some authors reporting no significant differences in the above-mentioned cytokines [46]. Such contrasting data are, at least in part, due to wide heterogeneity of samples in terms of severity, co-morbidities, anthropometrics, and clinical data.

## 5. Conclusions

In conclusion, our data suggest adiponectin as a possible serum biomarker in BCO. The functional role and molecular mechanisms of adiponectin regulation remain unknown, but an anti-inflammatory activity of adiponectin can be hypothesized. Indeed, in this scenario, the increased levels of adiponectin in the BCO cohort compared to those in COPD patients without bronchiectasis might be determined by their worse airway inflammatory state. Additional studies are warranted comparing bronchiectasis with and without COPD to define a panel of novel biomarkers in bronchiectasis that will facilitate classification and establish the progression of COPD disease. Whether adiponectin might contribute to developing novel therapeutic approaches in airway inflammation needs to be clarified.

## Figures and Tables

**Table 1:** Biochemical and clinical findings in study populations.

Parameter		COPD	BCO	Controls	*p*-Value
		n = 29	n = 30	n = 29	
Age, mean (SD)		60.7 (8.4)	60.0 (15.9)	56.6 (10.3)	0.37
Sex	F	13 (45%)	15 (50%)	15 (52%)	0.86
	M	16 (55%)	15 (50%)	14 (48%)	
Smoking Status	Current	13(44.8%)	7 (23.3%)	0 (0%)	<0.001
	Former	16(55.2%)	23 (76.7%)	0 (0%)	
	Ever	0 (0%)	0 (0%)	29 (100%)	
Pack Years, mean (SD)		27.4 (10.4)	26.8 (10.6)	-	0.82
BMI, mean (SD)		27.7 (5.0)	25.8 (4.2)	25.1 (2.3)	0.053
Total cholesterol, mean (SD)		177.9 (44.6)	174.5 (35.2)	200.0 (41.2)	0.043
Triglycerides, mean (SD)		122.0 (71.4)	106.2 (33.1)	127.1 (64.5)	0.36
Glycemia, mean (SD)		100.4 (23.2)	91.5 (14.5)	85.1 (14.5)	0.008
AST, mean (SD)		21.3 (10.2)	18.6 (6.0)	19.8 (5.3)	0.40
ALT, mean (SD)		20.2 (10.2)	20.3 (8.9)	20.8 (9.9)	0.97
WBC, mean (SD)		8.0 (2.1)	7.4 (2.4)	10.2 (17.2)	0.55
Neutrophils%, mean (SD)		62.6 (13.1)	59.3 (8.6)	57.6 (8.1)	0.24
Lymphocytes%, mean (SD)		27.0 (11.8)	29.5 (7.7)	32.8 (6.1)	0.077
NLR, mean (SD)		3.6 (4.8)	2.3 (1.3)	1.8 (0.5)	0.077
RBC, mean (SD)		4.64 (0.4)	4.86 (0.6)	4.7 (0.3)	0.174
HGB, mean (SD)		13.8 (1.1)	14.0 (1.7)	14.1 (1.0)	0.678
Adiponectin, mean (SD)		18.2 (1.3)	37.6 (8.3)	9.1 (5.6)	**<0.001**
FVC% predicted, mean (SD)		83.2 (25.8)	66.7 (17.8)	-	**<0.001**
FEV1% predicted, mean (SD)		67.3 (22.3)	52.3 (16.5)	-	**<0.001**
GOLD Stage					
1		6 (20.7)	4 (13.3)		0.684
2		11 (37.9)	11 (36.7)		0.866
3/4		12 (41.4)	15 (50)		0.687

**Table 2:** Multiple comparisons of adiponectin levels among the 3 groups: COPD, BCO, and controls.

Group		Difference Mean (I–J)	Standard Error	*p*-Value	Confidence Interval 95%	
					**Lower limit**	**Upper limit**
CBO vs.	Controls	28.51	1.53	**<0.001**	24.77	32.26
CBO vs.	COPD	19.39	1.53	**<0.001**	15.65	23.14
Controls vs.	COPD	−9.12	1.55	**<0.001**	−12.90	−5.35

**Table 3:** Correlations between adiponectin levels and main metabolic parameters.

	95% Wald’s Confidence Interval		Hypothesis Testing		
Parameter	Lower	Upper	Chi−Square Wald	Degree of Freedom (df)	*p*-Value
**BMI**	−0.65	0.84	0.06	1.00	0.80
**Glycemia**	−0.06	0.24	1.45	1.00	0.23
**Total Cholesterol**	−0.12	0.03	132	1.00	0.25
**Triglycerides**	−0.09	0.02	1.89	1.00	0.17

**Table 4:** Cytokine level comparison in COPD patients, BCO patients, and healthy subjects.

Cytokine (pg/mL)	COPD Patients (n = 29)	BCO Patients (n = 30)	Controls (n = 29)	*p*-Value
**IL-2**	8.12 ± 2.11	7.42 ± 1.26	15.72 ± 2.93	<0.001
**IL-4**	15.3 ± 1.81	11.83 ± 0.32	5.56 ± 1.15	<0.001
**IL-6**	9.56 ± 1.41	11.29 ± 3.11	7.71 ± 2.1	0.003
**IL-8**	58.03 ± 17.19	142.18 ± 30	20.48 ± 4.91	<0.001
**IL-10**	7.81 ± 1.77	6.54 ± 0.54	23.87 ± 3.73	<0.001
**IFN-γ**	17.21 ± 3.51	125.30 ± 25.89	13.25 ± 2.74	<0.001
**GM-CSF**	15.03 ± 3.02	94.99 ± 10.02	12.78 ± 2.21	<0.001

## Data Availability

Data are available upon request.

## Supplementary Materials

The following supporting information can be downloaded at: https://www.mdpi.com/article/10.3390/life13020444/s1, Figure S1: Adiponectin distribution among different airflow limitation stages in BCO patients. The airflow limitation group was categorized as (1) Mild; (2) Moderate; (3) Severe; Figure S2: Uncropped blot of Figure 1. Table S1: Sensitivity of each analyte measured.
