# Supplementary material for: Adiponectin Increase in Patients Affected by Chronic Obstructive Pulmonary Disease with Overlap of Bronchiectasis
Source: Life (Basel). 2023 Feb 4;13(2):444. doi: 10.3390/life13020444 (PMC9959162; doi:10.3390/life13020444)
Supplement: Supplementary file 1 [file life-13-00444-s001.zip › life-2110857-supplementary.pdf]

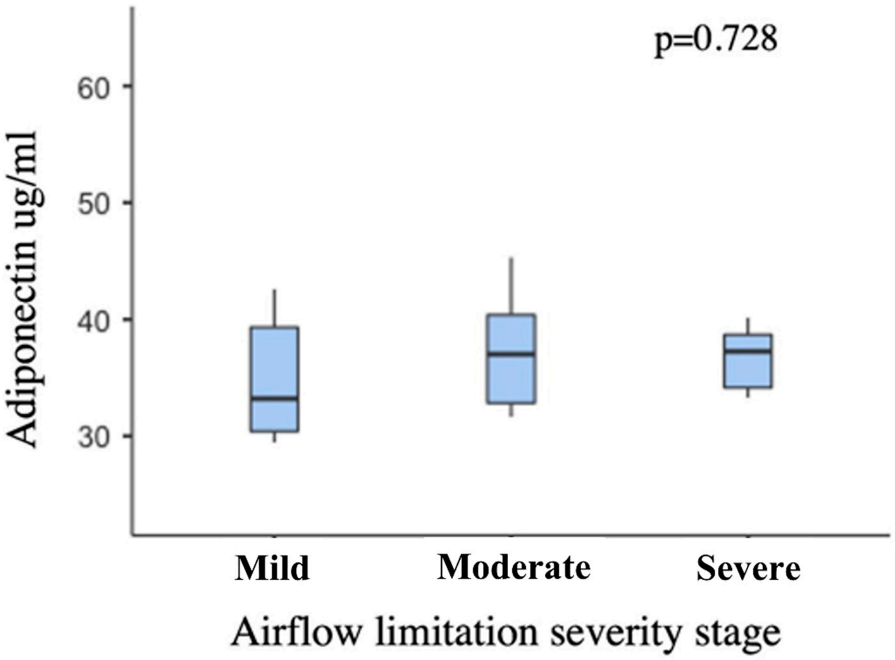

Figure S1. Adiponectin distribution among different airflow limitation stages in BCO patients. The airflow limitation group was categorized as 1) Mild; 2) Moderate; 3) Severe.

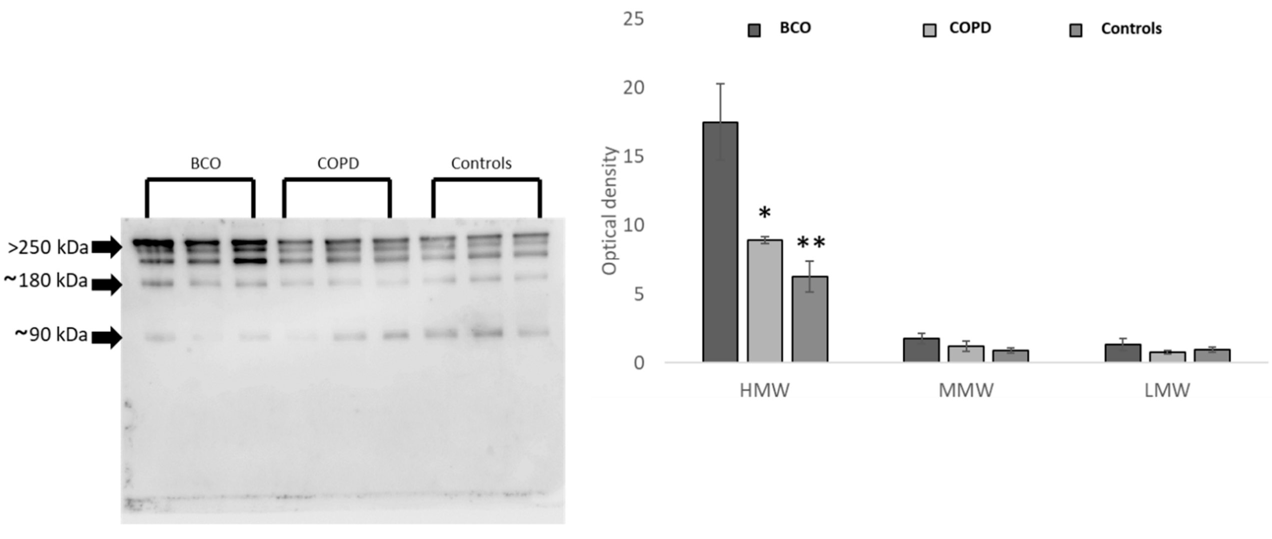

Figure S2. Uncropped blot of Figure 1. * $p < 0.05$, ** $p < 0.01$.

Targets	Assay Sensitivity (pg/ml)	Assay Precision	
		Intra-Assay %CV	***Inter-Assay %CV***
IL2	1.6	7	9
IL4	0.7	9	8
IL6	2.6	7	11
IL8	1.0	9	4
IL10	0.3	5	6
INFγ	6.4	15	9
GM-CSF	2.2	12	6

Supplementary Table S1. Sensitivity of each analyte measured.
